# Factors associated with community commitment among older adults: a stratified analysis of community group leaders and members

**DOI:** 10.1186/s12877-022-03361-4

**Published:** 2022-08-15

**Authors:** Hina Taguchi, Etsuko Tadaka, Yuka Iwata, Azusa Arimoto

**Affiliations:** 1 The University of Tokyo Hospital, 7-3-1 Hongo, Bunkyo-ku, Tokyo, 113-8655 Japan; 2 Department of Community and Public Health Nursing, Graduate School of Health Sciences and Faculty of Medicine, Hokkaido University, N12-W5, Kita-Ku, Sapporo, Hokkaido 060-0812 Japan; 3 Department of Community Health Nursing, Graduate School of Medicine, Yokohama City University, 3-9 Fukuura, Kanazawa-ku, Yokohama, Kanagawa 236-0004 Japan

**Keywords:** Community, Community commitment, Community group, Leader, Member, Older adults, Role

## Abstract

**Background:**

Community commitment through group activities in the community is associated with improved health outcomes in older adults and has a ripple effect on community development. However, factors associated with community commitment through group activities in the community have not been examined. The purpose of this study was to examine individual factors and group-related factors in association with community commitment among older adult leaders and members of community groups in Japan.

**Methods:**

We mailed self-administered questionnaires to all older adults participating in a community group (*N* = 1,898) in a ward of Yokohama city, the largest designated city in Japan. Variables included demographic characteristics, community commitment (Community Commitment Scale), individual factors, and group-related factors. We used logistic regression analysis to assess the association among study variables.

**Results:**

A total of 1,154 people completed the questionnaire. The valid response rate was 48.8%. Respondents’ mean age was 78.3 years (standard deviation [SD] = 6.1, range 65–100 years), 79.6% were women, 55.9% were married, and 10.0% were employed. Factors associated with community commitment among group leaders were scores for self-efficacy in the health promotion scale (SF-15; mean ± SD: 48.5 ± 7.1), 5-item World Health Organization Well-Being Index (mean ± SD: 17.9 ± 4.3), and Lubben Social Network Scale, Japanese version (mean ± SD: 19.5 ± 6.9), as well as a perception of deriving pleasure from group participation (mean ± SD: 91.2 ± 9.4). Factors associated with community commitment among group members were economic status (Sufficient; n [%]: 749 [85.9]), frequency of going out (mean ± SD: 5.1 ± 1.8), years of group participation (mean ± SD: 6.2 ± 5.0), and perceptions of their role in the group (Yes; n [%]: 254 [30.4]) as well as the above factors for leaders. A supplementary qualitative analysis of participants’ free-text responses extracted seven categories: community support, resource mobilization, partnership action, asset management, participatory decision-making, linkages and networking, and community dissemination, related to perception of a role in the group.

**Conclusion:**

Our results emphasize the importance of considering the different associations of community commitment through group activities in the community between group leaders and members, including the role of older adults in community groups, and suggest different approaches for group leaders and members.

## Background

Community commitment is a sense of community and refers to the psychological bond between community members and their community. Community commitment between a community member and a particular community arises from the member’s belief that their involvement in the community provides them with net benefits that are not easily available elsewhere [1]. The concept of commitment originates from the study of organizational behavior. Becker first defined organizational commitment as the behavioral intention to maintain an ongoing relationship between individuals and organizations [2]. Community commitment reflects an intention to maintain a long-term relationship between people and their communities.

Community commitment is an especially important resource for older adults [3, 4]. Community refers to human ecology and comprises connections among individuals, the members of groups, and society [5]. Therefore, a feeling of being part of a community, which can arise with community commitment, is one of the most basic human needs [6]. When this need goes unmet, it can not only serve as the basis for problems such as social isolation [7], crime, and accidents in the community [8–11], but serious impacts on the internal lives of older individuals, which are not visible from the outside, can occur, such as effects on their dignity. Additionally, a low sense of belonging together with low community commitment can result in declining health [12–15] and may even result in early death [16–18].

Older adults can become part of their community through community group activities [19, 20]. A number of longitudinal studies have shown that community group activities are associated with improved health outcomes in older people, including maintenance of instrumental activities of daily living [21] as well as cognitive and mental health [22], prevention of frailty progression [23], and promotion of healthy aging. These benefits can be generated in various types of community activities through community commitment [24]. Therefore, promoting effective community group activities among older people is an important public health policy [25, 26]. However, little is known about factors influencing community commitment in older populations.

For the development of community commitment in older adults through community group activities, two points should be considered. First, it is necessary to understand not only individual factors but also group-related factors. The operational definition of individual factors is modifiable physical, mental, and social characteristics among older adults. One study found that social cohesion was significantly associated with consistent participation in a walking group [27]. We defined social cohesion in the context of community commitment as attachment to or connection with the community to which an individual belongs that can be enhanced through participation in neighborhood activities. Social cohesion in this context is considered an individual factor because what is being captured is the sense of attachment, connectedness, and belonging that each individual holds with the community and community group to which they belong. The operational definition of group-related factors is modifiable, structural, and systematic factors in community group activities. Community is defined as a social group determined by geographic boundaries or common values and interests [28] and is considered to be individuals interacting with each other [29]. An approach involving interaction between leaders and members of a social group who live within the same geographic area and share the same common values and interests regarding longevity and health is more population-oriented, preventive, sustainable, and cost-effective than single-level approaches such as one-on-one treatments and interventions provided to individual older adults by doctors, nurses, and therapists [29]. Second, it is necessary to consider that the related factors differ between group leaders and group members [30, 31]. Community group activities for health promotion in Japan are nationally, institutionally, and culturally driven by local residents rather than by the government. Each community group has a skilled leader and a diverse group of community members. There is evidence regarding the characteristics of group members and leaders that are significantly related to the functioning of community group activities and promotion of their health [32, 33]. There may be mutual influence between leaders and members, who may have different backgrounds to begin with, in terms of social orientation, activity levels, and extent of interaction in the community [34]. However, there is no evidence of associations between community commitment and individual- and group-related factors among leaders and members who are older adults participating in community group activities.

## Methods

### Aim, design and setting of the study

The aim of our study was to examine the associations of community commitment with individual factors (defined as modifiable physical, mental, and social characteristics among older adults) and group-related factors (defined as modifiable, structural and systematic factors of community group activities) among older group leaders and members participating in community group activities. We conducted a cross-sectional study from October 23 to November 29, 2019, in a ward certified by Yokohama city, the largest designated city in Japan.

### Participants

This study comprised all 1,898 older adults belonging to a community group certified by the local government of Yokohama city, Japan. The criteria for including participants were as follows: (1) age 65 years and over, (2) belonging to a community group certified by the local government of Yokohama city in Japan, and (3) having the ability and willingness to complete the questionnaire.

In Yokohama city, Japan, older people are encouraged to gather independently and engage in health-promoting activities. In addition, support by local government officials is provided on a ward-by-ward basis. A ward here is an administrative district and refers to the basic geographic and local government area of the municipality in which an older resident is registered. Participants in such activities primarily focus on wellness and also serve as secondary resources in their community as they can promote their neighbors’ health behavior and community commitment. The division of roles between leaders and members is not based on a leader training program but rather is determined and managed by the residents themselves through discussions within the group. In the group activities, factors for residents’ own decision-making and management roles may vary depending on the composition and activities of the group. As our research subject, a community group involves a group of two or more older people who independently gather in the community and engage in health promotion activities at least once a month. The local government certifies these groups as community groups, and public health nurses and other administrative staff regularly monitor and support the group activities. Examples of specific activities are walking, engaging in exercises, choral singing, and cooking.

**Fig. 1:**
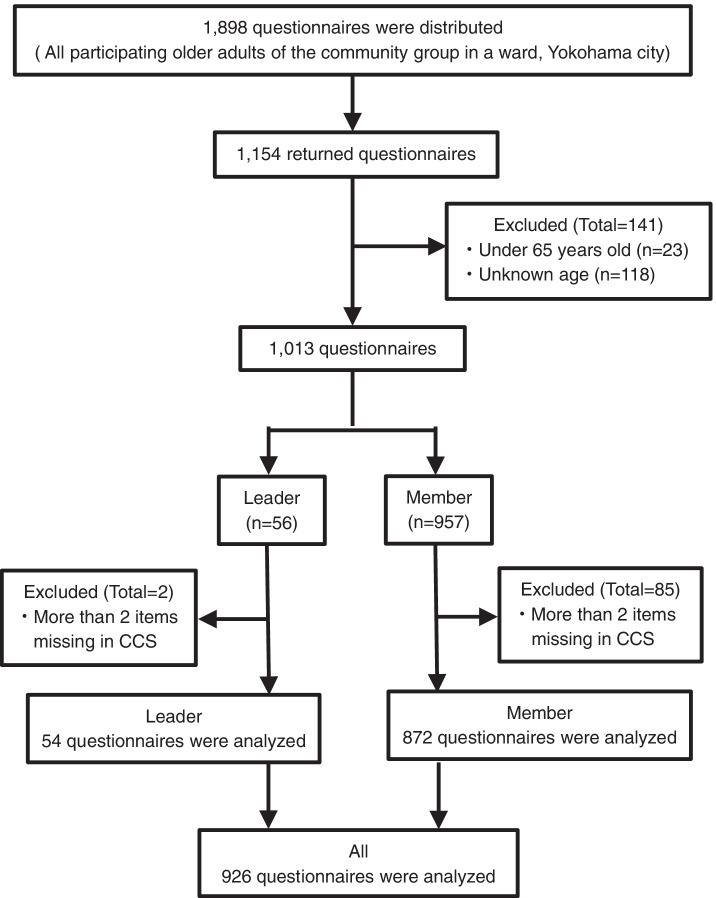
Subject flow diagram

Self-administered questionnaires were mailed to all participants of a community group. In total, 1,154 people responded (response rate 60.8%). Of these, 228 people were excluded based on the exclusion criteria: (1) under 65 years old or unknown age (*n* = 141), and (2) two or more missing items on the Community Commitment Scale (CCS) (*n* = 87). To enroll sufficient participants in each group, we included responses with one missing item in the CCS; missing items were replaced with the leader or member population mean. The mean was used because we assumed that there would be differences between leaders and members in CCS scores. Thus, 926 older adults comprised the final sample in the analysis (valid response rate 48.8%) (Fig. 1).

## Measurements

### Demographic characteristics

Participants’ characteristics included age, sex, living arrangements (living alone/with a spouse/with children/with a spouse and children/with children and grandchildren/other), marital status (married/divorced or widowed/unmarried), time of living in the ward (years), being employed (yes/no), economic status (subjectively sufficient = 0, subjectively insufficient = 1), visual function (visual impairment/none), and hearing function (hearing impairment/none). Regarding economic status, participants were asked “Do you have any concerns about your financial situation?” The frequency of going out was assessed using the number of days per week. The purpose of going out was not asked. When participants left their home, they were considered to be “going out.” If a participant went outside the home at least once a day, this was counted as one day of going out. Whether respondents were currently under treatment for any diseases was confirmed using the Health Statistics of the Number of Individuals Receiving Medical Treatment for Older People in Japan (yes/no) and certification of the need for long-term care under the Long-Term Care Insurance system in Japan (Support need levels 1 and 2 and Care need levels 1 to 5; a larger number indicates a greater need for care). Group-related characteristics among participants included: years of group participation, the trigger for group participation, purpose of group participation, and transportation to group activities.

### Dependent variable

The Community Commitment Scale (CCS) is a scale used to assess sense of community, which was developed by considering the cultural diversity of Japanese people who value relationships with neighbors and communities but who inhabit a limited land area on the Japanese islands [35]. Previous studies in Japan [36] and Korea [37] have demonstrated that the CCS can be used to psychometrically measure older adults’ socialization and sense of belonging via the strength of their community networks and that the CCS is a useful indicator to monitor older adults living in the community who may be socially isolated. The CCS has a Cronbach’s alpha (which conveys the internal consistency of the scale) of 0.75 in local volunteers and 0.78 in general residents. The correlation coefficients between scores of the CCS and the Brief Sense of Community Scale [38] were 0.54 for local volunteers and 0.62 for general residents. This scale comprises eight items, four each in the subscales of socializing and belonging. Each item is scored on a four-point scale, as follows: 0 (not confident at all), 1 (not confident slightly), 2 (confident slightly), and 3 (absolutely confident). Higher scores (range 0–24) indicate a higher degree of community commitment. No cutoff point for the CCS has been determined. We set a cutoff point for the CCS based on the distribution of our study population.

### Independent variables

#### Individual factors

Cognitive function was measured using the self-administered dementia checklist (SDC) [39, 40]. The SDC was developed to enable community-dwelling older people to recognize their declining functions at an early stage of dementia progression. This scale includes 10 items, each using a 4-point Likert scale, and consists of two subscales: subjective cognitive decline (5 items) and instrumental activities of daily living (5 items); both constructs have been shown to predict dementia onset. Scores range from 10 to 40, with higher scores indicating a higher risk of cognitive function decline.

Health behavior was measured using self-efficacy in the health promotion scale among community-dwelling older people (SF-15) [41]. The SF-15 was developed to predict the implementation of appropriate health promotion behaviors according to an older person’s health status and demographic characteristics. This scale includes 15 items comprising a wide range of health behaviors, such as weight control and tooth brushing, as well as health behaviors that make use of community resources and systems, each using a 4-point Likert scale. Scores range from 15 to 60, with higher scores indicating a higher self-efficacy in health care among older adults. The Cronbach’s alpha is 0.93.

Mental health was measured using the five-item World Health Organization Well-Being Index (WHO-5) [42]. This scale includes five items: 1) I have felt cheerful and in good spirits, 2) I have felt calm and relaxed, 3) I have felt active and vigorous, 4) I woke up feeling fresh and rested, and 5) My daily life has been filled with things that interest me, each using a 6-point Likert scale. Scores range from 0 to 25, with higher scores indicating higher levels of current mental health.

Social networks were measured using the Lubben Social Network Scale, Japanese version (LSNS-6) [43]. This scale includes six items that measure the size, closeness, and frequency of contact with each respondent’s social network of family and friends, each using a 6-point Likert scale. Scores range from 0 to 30, with higher scores indicating a greater social network of family and friendships.

#### Group-related factors

Years of group participation were assessed using the following question “How many years have you participated in the group?”.

A perception of having a role in the group was assessed using the following question, “Do you have some roles in your group activities?” (yes/no). For participants who answered yes, we asked them to provide free-text responses describing the role they play in the group.

The perception of deriving pleasure from group participation was assessed using a visual analog scale. This scale comprises a 10 cm line with descriptive anchors at each end, ranging from “Not fun at all” to “Very fun.” Scores range from 0 to 100, with higher scores indicating greater pleasure.

### Statistical analysis

The analyses were stratified according to roles as group leader or group member because it is highly likely that each leader/member has different factors of community commitment. Descriptive analyses included frequency and percentage for categorical variables and mean (standard deviation, SD) for continuous variables. Differences in the CCS and independent variables were compared with the *t*-test and *χ*^2^ test. Logistic regression analysis was performed to assess key determinants of the CCS. In single-factor logistic regression analysis, adjustment for covariates was conducted for age and sex. The results were considered statistically significant with *p* < 0.05 or if the 95% confidence interval (CI) did not include 1. IBM SPSS software, version 28.0 (IBM Corp., Armonk, NY, USA) was used for the analysis.

We also conducted a supplementary qualitative analysis of the open-ended responses regarding perception of the role in the group. From the open-ended data, we identified categories according to similarities and differences [44].

### Ethical considerations

Participants were informed, both in writing and verbally, of the purpose and methods of the study and that there would be no disadvantage if they withdrew or refused to participate in the study. Participants were informed that study participation was voluntary and that completing and returning the questionnaire indicated their consent to participate in the study. This study was approved by the Institutional Ethical Review Board of the School of Medicine, Yokohama City University (No. A201200008).

## Results

### Demographic characteristics

Table 1 show the demographic characteristics of participants. Respondents’ mean age was 78.3 years (SD = 6.1, range 65–100 years), 79.6% were women, 35.9% lived with a spouse, 55.9% were married, and 10.0% were employed. Regarding the trigger of group participation, 45.7% responded that they were invited by a friend; regarding the purpose of group participation, 74.7% answered to keep fit; and regarding transportation to group activities, 92.2% of respondents said on foot (Table 2).

**Table 1:** Demographic characteristics of the study participants

	Total (*n* = 926)		Leader (*n* = 54)		Member (*n* = 872)	
	n or mean	% or (SD)	n or mean	% or (SD)	n or mean	% or (SD)
Age	78.3	(6.1)	76.7	(5.0)	78.4	(6.1)
Sex						
Female	737	79.6	33	61.1	704	80.7
Missing	15	1.6	2	3.7	13	1.5
Living arrangements						
Living alone	264	28.5	11	20.4	253	29.0
Living with spouse	332	35.9	28	51.9	304	34.9
Living with children	136	14.7	5	9.3	131	15.0
Living with spouse and children	122	13.2	7	12.9	115	13.2
Living with children and grandchild	47	5.1	3	5.5	44	5.0
Others	22	2.3	0	0.0	22	2.6
Missing	3	0.3	0	0.0	3	0.3
Time of living in the ward	42.9	(17.8)	44.9	(18.6)	42.8	(17.8)
Being employed						
Yes	93	10.0	5	9.3	88	10.1
Missing	17	1.8	0	0.0	17	1.9
Economic status						
Sufficient	800	86.4	51	94.4	749	85.9
Insufficient	120	13.0	3	5.6	117	13.4
Missing	6	0.6	0	0.0	6	0.7
The frequency of going out	5.2	(1.7)	5.4	(1.5)	5.1	(1.8)
Under medical treatment						
None	169	18.3	11	20.4	158	18.1
Hypertension	450	48.6	23	42.6	427	49.0
Arthralgia	185	20.0	9	16.7	176	20.2
Diabetes	165	17.8	6	11.1	159	18.2
Cataract	142	15.3	5	9.3	137	15.7
Certification for long-term care independence	805	86.9	50	92.6	755	86.6

Under medical treatment was asked for multiple responses

**Table 2:** Demographic characteristics of the study community groups

	Total (*n* = 926)		Leader (*n* = 54)		Member (*n* = 872)	
	n or mean	% or (SD)	n or mean	% or (SD)	n or mean	% or (SD)
The trigger for group participation						
Invitation from friends	423	45.7	7	13.0	416	47.7
Invitation from staff member of community association	396	42.8	16	29.6	380	43.6
Invitation from neighborhood administrative staff	67	7.2	13	24.1	54	6.2
Seeing publicity	78	8.4	5	9.3	73	8.4
Other	74	8.0	16	29.6	58	6.7
Purpose of group participation						
Keep fit	692	74.7	44	81.5	648	74.3
Interaction	615	66.4	33	61.1	582	66.7
Activity description	236	25.5	15	27.8	221	25.3
Getting to know neighborhood administrative staff	95	10.3	16	29.6	79	9.1
Other	23	2.5	6	11.1	17	1.9
Transportation to group activities						
On foot	854	92.2	50	92.6	804	92.2
Bus	79	8.5	4	7.4	75	8.6
Car	28	3.0	2	3.7	26	3.0
Bicycle	19	2.1	1	1.9	18	2.1
Train	15	1.6	0	0.0	15	1.7
Other	17	1.8	0	0.0	17	1.9

Trigger of group participating, Purpose of group participation, and Transportation to group activities were asked for multiple responses

### Dependent variable

Table 3 shows the CCS scores. Both the mean and median CCS scores were one point higher among leaders than members. Moreover, both skewness and kurtosis for leaders and members did not show a normal distribution, suggesting that the scores were very high for a small number of leaders and members, respectively. From the above, we decided that it was appropriate to dichotomize the groups in subsequent multiple logistic analysis. The mean ± SD (range) of CCS scores was 17.2 ± 3.7 (7–24) for the total participants, 18.4 ± 3.2 (11–24) for leaders, and 17.2 ± 3.8 (7–24) for members. We set a cutoff of 17/18 for the overall analysis, 18/19 for the analysis of leaders, and 17/18 for the analysis of members, based on the mean CCS scores: 17.2 (total; *n* = 926), 18.4 (leaders; *n* = 54), and 17.2 (members; *n* = 872) among study participants (Table 3).

**Table 3:** Univariate analysis of the community commitment

	All (*n* = 926)	Leader (*n* = 54)	Member (*n* = 872)
Mean (SD)	17.2 (3.7)	18.4 (3.2)	17.2 (3.8)
Median	17.0	18.0	17.0
Mode	18.0	17.0	18.0
Range	17.0	13.0	17.0
Skewness	-0.1	-0.1	-0.1
Kurtosis	-0.7	-0.5	-0.7

### Independent variables

Table 4 lists the related factors in univariate analysis (independent variables) of the CCS. The demographic characteristics with significant differences in the CCS were sex (*p* < 0.05), years of living in the wards (*p* < 0.01), economic status (*p* < 0.01), frequency of going out (*p* < 0.01), trigger of group participation (invitation from staff member of community association/others) (*p* < 0.05), purpose of participation (interaction/getting to know neighborhood administrative staff/activity description) (*p* < 0.05) (Tables 1 and 2). Individual factors showing significant differences with CCS scores were scores on the SF-15 (*p* < 0.001), and WHO-5 (*p* < 0.001); LSNS-6 (*p* < 0.001); and frequency of going out (*p* < 0.01) (Table 4). Group-related factors showing significant differences with CCS scores were perception of role in the group (*p* < 0.05), years of group participation (*p* < 0.05), and perception of pleasure from the group (*p* < 0.001). Based on the above, considering multicollinearity, 8 of the 15 factors were used as independent variables, and age and sex were entered into the final multivariate logistic regression analysis as control variables.

**Table 4:** Univariate analysis of related factors of the community commitment

	All (*n* = 926)			Leader (*n* = 54)			Member (*n* = 872)		
	n or mean	% or (SD)	*p*-value	n or mean	% or (SD)	*p*-value	n or mean	% or (SD)	*p*-value
Sex									
Female	737	79.6	0.013	33	61.1	0.477	704	80.7	0.002
Missing	15	1.6		2	3.7		13	1.5	
SDC									
High (17 points or less)	561	60.6	0.460	41	75.9	0.255	520	59.6	0.721
Low (18 points or more)	299	32.3		13	24.1		286	32.8	
Missing	66	7.1		0	0.0		66	7.6	
Perceptions of role in group									
Existence	305	34.3	0.024	51	94.4	0.415	254	30.4	0.044
Missing	37	4.0		0	0.0		37	4.2	
Age	78.3	(6.1)	0.278	76.7	(5.0)	0.718	78.4	(6.1)	0.263
Economic status									
Sufficient	800	86.4	0.002	51	94.4	0.415	749	85.9	0.002
Insufficient	120	13.0		3	5.6		117	13.4	
Missing	6	0.6		0	0.0		6	0.7	
SF-15	46.8	(7.8)	< 0.001	48.5	(7.1)	0.016	46.7	(7.9)	< 0.001
WHO-5	16.3	(5.1)	< 0.001	17.9	(4.3)	0.011	16.2	(5.1)	< 0.001
LSNS-6	17.5	(5.9)	< 0.001	19.5	(6.9)	0.001	17.4	(5.8)	< 0.001
Frequency of going out	5.2	(1.7)	0.009	5.4	(1.5)	0.223	5.1	(1.8)	0.022
Years of group participation	6.4	(5.1)	0.018	9.6	(6.0)	0.658	6.2	(5.0)	0.034
Perceptions of pleasure in group	82.8	(17.2)	< 0.001	91.2	(9.4)	0.017	82.3	(17.5)	< 0.001

Frequency of going out, (days/week)

Years of group participation, (years)

Perceptions of pleasure in group, Visual analogue scales. (0 − 100.0)

*SD* Standard deviation, *SDC* Self-administered dementia checklist, *SF-15* “self-efficacy for health promotion scale” in community-dwelling elderly (15.0 − 60.0), *WHO-5* WHO-5 Japanese version (0 − 25.0), *LSNS-6* Lubben Social Network Scale (0 − 30.0)

### Factors related to CCS scores

Table 5 shows the related factors in multiple logistic analysis of the CCS. Factors that were associated with CCS scores were economic status (odds ratio [OR]: 0.57, 95% CI: 0.38–0.85); scores on the SF-15 (OR: 1.08, 95% CI: 1.06–1.10), WHO-5 (OR: 1.11, 95% CI: 1.08–1.15), and LSNS-6 (OR: 1.08, 95% CI: 1.06–1.11); and frequency of going out (OR: 1.11, 95% CI: 1.03–1.20), years of participating in the group (OR: 1.03, 95% CI: 1.00–1.06), perception of a role in the group (OR: 1.42, 95% CI: 1.05–1.92), and perception of pleasure from group participation (OR: 1.03, 95% CI: 1.03–1.04) (Table 5).

**Table 5:** Multiple logistic analysis of related factors of the community commitment

	All (*n* = 926)			Leader (*n* = 54)			Member (*n* = 872)		
Items	β	*P* value	OR (95% CI)	β	*P* value	OR (95% CI)	β	*P* value	OR (95% CI)
Economic status	-0.57	**	0.57 (0.38–0.85)	−	−	−	-0.60	**	0.55 (0.36–0.83)
SF-15	0.07	***	1.08 (1.06–1.10)	0.09	*	1.10 (1.00–1.20)	0.07	***	1.08 (1.05–1.10)
WHO-5	0.11	***	1.11 (1.08–1.15)	0.18	*	1.19 (1.02–1.39)	0.11	***	1.11 (1.08–1.15)
LSNS-6	0.08	***	1.08 (1.06–1.11)	0.16	**	1.18 (1.04–1.33)	0.07	***	1.08 (1.05–1.10)
Frequency of going out	0.10	**	1.11 (1.03–1.20)	−	−	−	0.10	*	1.10 (1.02–1.19)
Years of group participation	0.03	n.s	1.03 (1.00–1.06)	−	−	−	0.03	n.s	1.03 (1.00–1.06)
Perceptions of role in group	0.35	*	1.42 (1.05–1.92)	−	−	−	0.37	*	1.44 (1.07–1.96)
Perceptions of pleasure in group	0.03	***	1.03 (1.03–1.04)	0.09	*	1.10 (1.02–1.18)	0.03	***	1.03 (1.02–1.04)

Analysis of All was adjusted by age, sex, and leader versus participant

Analysis of Leader and Member were adjusted by age and sex

Economic status, (insufficient = 1, sufficient = 0)

SF-15, “self-efficacy for health promotion scale” in community-dwelling elderly

WHO-5, WHO-5 Japanese version

LSNS-6, Lubben Social Network Scale

Frequency of going out, "How many days do you go out?" (days/week)

Years of group participation, “How many years have you been participated in the group?” (years)

Perceptions of role in group, “Do you have some roles in your group activities?” (Yes = 1, No = 0)

Perceptions of pleasure in group, visual analogue scales

*n.s* Not significant

^*^p < 0.05; **p < 0.01; ***p < 0.001

There were differences between leaders and members in the associations of independent variables with CCS scores (Table 5). Among leaders, those with higher SF-15 scores were 1.10 times (95% CI: 1.00–1.20) more likely to have higher CCS scores; those with higher WHO-5 scores were 1.19 times (95% CI: 1.02–1.39) more likely to have higher CCS scores; those with a larger social network were 1.18 times (95% CI: 1.04–1.33) more likely to have higher CCS scores, and leaders with greater perception of pleasure from group participation were 1.10 times (95% CI: 1.02–1.18) more likely to have higher CCS scores. Members showed differences in economic status, frequency of going out, and perception of role in the group, in addition to the above factors identified for leaders. Among members, those with lower economic status were 0.55 times (95% CI: 0.36–0.83) more likely to have lower CCS scores, those with a high frequency of going out were 1.10 times (95% CI: 1.02–1.19) more likely to have higher CCS scores, and those with more positive perceptions of their role in the group were 1.44 times (95% CI: 1.07–1.96) more likely to have higher CCS scores (Table 5). Supplementary qualitative analysis yielded seven categories of role cognitive content among older adults involved in group activities: community support, resource mobilization, partnership action, assets management, participatory decision-making, linkages and networking, and community dissemination (Table 6).

**Table 6:** Qualitative data for perceptions of roles in groups

Category	Code
Community support	“I’m on cleaning duty, which is assigned to us on a rotating basis.” (M, Female, aged 86 years)
	“I am in charge of arranging lunches, preparing tea, and making cakes for birthday celebrations.” (M, Female, aged 77 years)
	“I prepare the items to be used and book the venue.” (M, Female, aged 79 years)
Resource mobilization	“I am the liaison between members of the group and staff of the ward office.” (L, Male, aged 67 years)
	“I reach out and call people in my community.” (M, Female, aged 83 years)
Partnership action	“I run the group.” (L, Female, aged 75 years)
	“I assist in group management as a sub-leader.” (M, Male, aged 71 years)
Asset management	“I am in charge of budget management and execution as a treasurer.” (M, Female, aged 71 years)
Participatory decision-making	“My role is to participate.” (M, Female, aged 79 years)
	“I think it is important to participate. I try to participate actively.” (M, Male, aged 85 years)
Linkages and networking	“I care for everyone so that they can have a good time." (L, Male, aged 71 years)
	“To look after the members.” (L, Female, aged 71 years)
	“Interact with local people in a harmonious way.” (M, Female, aged 77 years)
Community dissemination	“I’m trying to get as many people as possible to join and invite their friends.” (M, Female, aged 79 years)
	“I publicize our activities to the community.” (M, Male, aged 86 years)

*N* = 926, *L* Leader, *M* Member

## Discussion

Greater community commitment among participants through engagement in community groups would mean an increase in the absolute number of older adults in the community as a whole who are committed to the community. Community commitment via participation in community groups is associated with improved health outcomes in older adults and has a ripple effect on development of the community. However, factors associated with community commitment have not been examined. To the best of our knowledge, this was the first study to examine the associations of community commitment with individual factors and group-related factors among older adults participating as leaders or members in community groups. One clinical or policy implication of this study is the focus on and expansion of the “community” through the “older adult group” as a subject, purpose, and method in geriatrics, based on factors related to the CCS for both community group leaders and members, with the aim to increase healthy longevity. The participants in this study were representative of community-dwelling older adults who participate in community groups in urban areas. The mean age was 78.3 years for the total population, 76.7 years for leaders, and 78.4 years for members. Fewer leaders than members were under medical treatment for all items, which is inconsistent with previous studies on differences between group leaders and members [34]. In a previous study, African Americans aged 18 years and older were the target population, but there was no report of leader/member-specific group activities for older adults only. Because of differences in the target population, the results of the present study may not be consistent with those of the above study, and further research is needed.

The mean CCS score was 17.2 for the total population, 18.4 for leaders, and 17.2 for members. It has been reported that the mean CCS score among older people over 65 years old living in urban areas is 14.5 [45]. Our study population participated in regular group activities in the community, and they had higher CCS scores than the general population of older adults. Moreover, because leaders are required to collaborate with various local organizations in the management of group activities, they may have wider connections with the community and greater community orientation than group members. This result may be related to the fact that members of the general population are expected to be the primary resource in the community whereas leaders are expected to serve as advanced resources in community health networks and organizations [36].

Whether leader or member, we found an association of CCS scores with SF-15, WHO-5, LSNS-6 scores and a perception of deriving pleasure from group participation. As for the relationship between the SF-15 and CCS, there are reports that the greater the sense of belonging to a community, the better the health behavior of individuals [46–48]. The SF-15 scale encompasses not only self-efficacy in weight control and medication management but also in utilization of community resources, such as knowing about community resources and being able to consult with local professionals when in need [41]. Improving comprehensive health behaviors through health-promoting group activities, which is one objective of group activities, may also be important in increasing community commitment. As for the relationship between mental health (WHO-5) and the CCS, it has been pointed out that an increased sense of belonging to a community via community participation, rather than mere participation in community activities, is associated with mental health among older people [49, 50]. As for the relationship between social network (LSNS-6) and the CCS, previous studies have reported that talking frequently with neighbors was significantly related to both the desire to participate in community activities and the level of community commitment [51]. Studies in urban areas of Canada have shown a significant association between a sense of belonging to a community and social capital, such as having family and friends, which is consistent with our study [52]. As for the relationship between the perception of pleasure from group participation and the CCS, it can be said that positive perceptions of community activities and the community may increase owing to the pleasure derived from group activities in the community [53, 54]. If participants perceive pleasure from community-based group activities, this may promote greater social interaction and health-promoting behaviors as well as lead to greater commitment to the community [55, 56].

Sufficient economic status, frequency of going out, years of group participation, and perception of a role in the group were significantly associated with high CCS scores for members but not for leaders. The number (percentage) of respondents who reported sufficient economic status was 51 (94.4%) for leaders and 749 (85.9%) for members (Table 1). The mean ± SD of the frequency of going out was 5.4 ± 1.5 days for leaders and 5.1 ± 1.8 days for members (Table 3). As for sufficient economic status and frequency of going out, it is possible that a ceiling effect occurred in leaders, such that only members showed a significant association. Poor economic conditions can lead to limited social activity and declining health [57], which can also reduce interaction with and activity in the community. As for members with a lower economic situation and frequency of going out, CCS scores may also be decreased. An individualized approach tailored to the physical and living conditions of people with lower socioeconomic status may be necessary. Community groups can be an opportunity for older adults to secure regular opportunities to go out within the community and at low cost. In supporting community group activities, it may be necessary to consider the cost burden and ease of access for group participants.

One outstanding factor in this study was the relationship between role in the group and community commitment. Regarding roles, it has been reported that work and family roles are important to one's identity at older ages [58]. A previous study among older adults reported that taking on a leadership position in an organization can reduce the risk of dementia by approximately 20% in young-old people [59]. Other previous studies of geriatric residents have shown that high levels of social participation and having an important role in an organization have a protective effect against depressive symptoms in women and that there is an interaction between social participation, status in an organization, and rural residence among men [60]. It is also possible that members, who make up most participants in group activities, can enhance their sense of belonging to the community through their roles in group activities. Therefore, it is important for older people to become involved in group activities so that participants, especially members, can find role to increase community commitment among participants and enhance the wider effect on the community.

The supplementary qualitative analysis yielded seven categories of role cognitive content among older participants in group activities. These categories will be helpful in developing policies for group activities. The categories were interpreted identically for leaders and members, but there were some differences in the code. For example, resource mobilization was answered with respect to ward officials by leaders and with respect to community members by members. For working cooperatively with the local community surrounding the activity, this result was consistent with those reported in previous studies [31]. Additionally, participatory decision-making was only identified among members. This may have occurred owing to the likelihood that leaders easily came up with roles related to leadership tasks. In this study, the CCS score was approximately 1.5 times higher for the total population and approximately 1.4 times higher for members who answered “yes” to perception of a role in the group. Whereas there are reports on the importance of a group role [58, 59], there are others reports cautioning against making such roles too burdensome for participants [31]. Thus, to increase community commitment, apart from thinking of leaders as advanced resources for the community, it is important to appropriate their roles so that group participants can better recognize their own roles.

## Limitations

This study has several limitations. First, owing to the cross-sectional design, we could not establish a causal relationship between CCS scores and individual and group-related factors. Therefore, further longitudinal, and interventional studies are needed looking into the types of activities and types of roles and observing what occurs in each group activity via observation or conversation analyses [61] to add richness to the present findings. Second, the target population was limited to one urban area of Yokohama, Japan. It is unclear whether the results in Yokohama city can be generalized to suburban and rural areas; therefore, research in a wider range of areas are needed. Third, we did not fully capture information about older participants’ sociability outside of their community groups (i.e., whether they were relatively social individuals or whether they made any new friends in these community groups). Future studies should consider the level of social engagement possessed by older adults in the community as well as in community group activities.

## Conclusion

We examined the relationship between CCS scores and individual and group-related factors among older adults who were leaders and members in urban community groups. Our results emphasize the importance of considering the different associations of community commitment through group activities in the community between group leaders and members, including of the role of older adults in community groups, and suggest different approaches for group leaders and members.

## Data Availability

The datasets generated and analyzed during the current study are not publicly available because the Ethical Guidelines for Epidemiological Research by the Japanese Government and the National Basic Resident Registration System administered by the Ministry of Internal Affairs and Communications in Japan prohibit researchers from providing their research data to other third-party individuals but are available from the corresponding author on reasonable request.

## Abbreviations

CCS: The Community Commitment Scale
SDC: Cognitive function was measured using the self-administered dementia checklist
SF-15: The self-efficacy for health promotion scale in community-dwelling older people
WHO-5: The 5-item World Health Organization Well-Being Index
LSNS-6: The Lubben Social Network Scale, Japanese version
